# Ultrasound imaging versus morphopathology in cardiovascular diseases. Myocardial cell damage

**DOI:** 10.1186/1476-7120-3-32

**Published:** 2005-10-06

**Authors:** Giorgio Baroldi, Riccardo Bigi, Lauro Cortigiani

**Affiliations:** 1 Institute of Clinical Physiology, National Research Council, Milan and Pisa, Italy; 2 Cardiology, University School of Medicine and "A. De Gasperis" Foundation, Niguarda Hospital, Milan, Italy; 3 Cardiovascular Unit, "Campo di Marte" Hospital, Lucca, Italy

## Abstract

This review article summarizes the results of histopathological and clinical imaging studies to assess myocardial necrosis in humans. Different histopathological features of myocardial cell necrosis are reviewed. In addition, the present role of echocardiographic techniques in assessing irreversible myocardial damage is briefly summarized.

## 

By myocardial cell damage we mean a primary damage of the myocardial cell. In fact, the myocardium includes several other structures as vessels (arteries, veins, lymphatics), nerves, collagen matrix, interstitium, which can be primarily altered with subsequent secondary damage of myocardial cells. In general, both clinicians and pathologists believe in a unique pattern of myocardial necrosis due to ischemia; less frequently to inflammatory processes or rarely to storage diseases. In reality, three types of myocardial cell necrosis can be recognized [1-4] in relation to contraction cycle.

The myocardial cell may irreversibly arrest in: 1. relaxation 2. contraction 3. after progressive failure.

**1. **In the first condition the early histologic pattern is characterized by mild eosinophilia, increased length of sarcomeres and elongation of nuclei. This myocellular stretching is due to the action of intraventricular pressure on these elements in flaccid paralysis and visible within 30 minutes. The lesion is pathognomonic for myocardial infarct with its sequelae, namely a polymorphonuclear leukocytic infiltration which starts after 6–8 hours and disappears within 5 days, centripetal removal of necrotic tissue by macrophages and substitution by collagen ending in acellular and avascular, dense, scar (Fig. 1). No repair by granulation tissue is observed. In humans the infarct is monofocal and its size ranges from less than 10% to more than 50% of the total left ventricular mass. Erroneously named "coagulation necrosis" (coagulation never occurs), is better defined as *infarct *or *ischemic necrosis*. In contrast to the current belief oriented to reduce or avoid expansion of an infarct, death due to a myocardial infarct is not related to its size. About half of these cases have a size less than 20% of the left ventricular mass (Tab. 1). The same table show that: a) infarct size is not related to severity of coronary atherosclerotic lumen reduction and number of main vessels with sever stenosis; b) long survival (interval from the beginning to death) prevails in large infarcts; c) extensive myocardial fibrosis, as expression of chronic disease, does not correlate with infarct size; and d) the frequency of an occlusive thrombus is significantly higher in the largest infarcts supporting its secondary formation [5].

**Figure 1:**
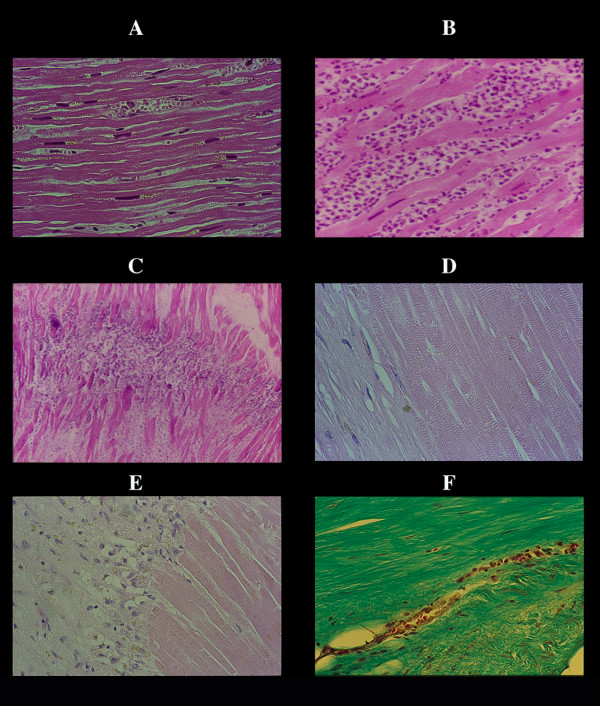
Infarct necrosis. The first change is lost of contraction with stretching of the myocardium in flaccid paralysis, resulting in a very early elongation of sarcomeres and nuclei (A) already visible within 30 minutes in experimental infarction. B, polymorphonuclear leukocyte infiltration from the periphery of the infarct after 6–8 hours. In the largest infarcts this infiltration arrests, along a line (maximal myocardial stretching in central part of infarct?) with occasional abscess-like formation (C). This infiltration disappears by lysis of the leukocytes, without evidence of myocellular colliquation or destruction (D). The myocardial cells maintain they sarcomeric registered order even in terminal healing phase. The repair process is carried out by macrophagic digestion (E) – and not by granulation tissue – ending in a compact and dense scar (F).

**Table 1:** Acute myocardial infarct size (% left ventricular mass) versus coronary atherosclerotic obstruction, extensive myocardial fibrosis (> 20% LVM), occlusive thrombus, survival and death in 200 selected cases*

**Infarct size %**	**N. cases**	**Coronary stenosis %**					**Extensive fibrosis**	**Survival days**			**Occlusive thrombus**
		**<69**	**≥ 70**	**1**	**2**	≥ 3**		**< 2**	**3–10**	**11–30**	
			
≤ 20	97	7	90	39	37	14	97	45	26	25	24
> 20	103	10	93	38	34	21	103	26	48	30	58
Total	200	17	183	77	71	35	200	71	74	55	82

*No invasive diagnostic/therapeutic techniques, no fibrinolysis.

** Number of main arteries with severe stenosis (≥ 70)

**2. **The opposite pattern is seen when the myocytes stop in contraction or better in hypercontraction (Fig. 2). In less than 10 minutes the hypercontracted myocardial cells break down forming hypereosinophilic transverse bands constituted by hypercontracted, extremely short sarcomeres with highly thickened Z lines. This rhexis of the myofibrillar apparatus ends in coagulation of sarcomeres, till a total, granular disruption. The sarcolemmal membrane is preserved and penetrated by macrophages which digest the necrotic material leaving empty sarcolemmal tubes ("alveolar" pattern) which subsequently collagenized (Fig. 3). These changes suggest that the mechanical contraction of the normal surrounding myocardium causes the break of these rigid elements in tetanic paralysis. This lesion is plurifocal with foci formed by one or few cells to thousands and is the typical necrosis obtained experimentally by catecholamine infusion and present in the myocardium of patients with pheochromocytoma. It must be stressed that in these experimental and human conditions no infarct necrosis is seen. Variously called as, microinfarct, infarct-like, focal myocytolysis, Zenker necrosis, coagulative myocytolysis, myofibrillar degeneration, focal myocarditis and overall contraction band necrosis, the more appropriate term is *catecholamine necrosis *to indicate cause-effect relationship.

**Figure 2:**
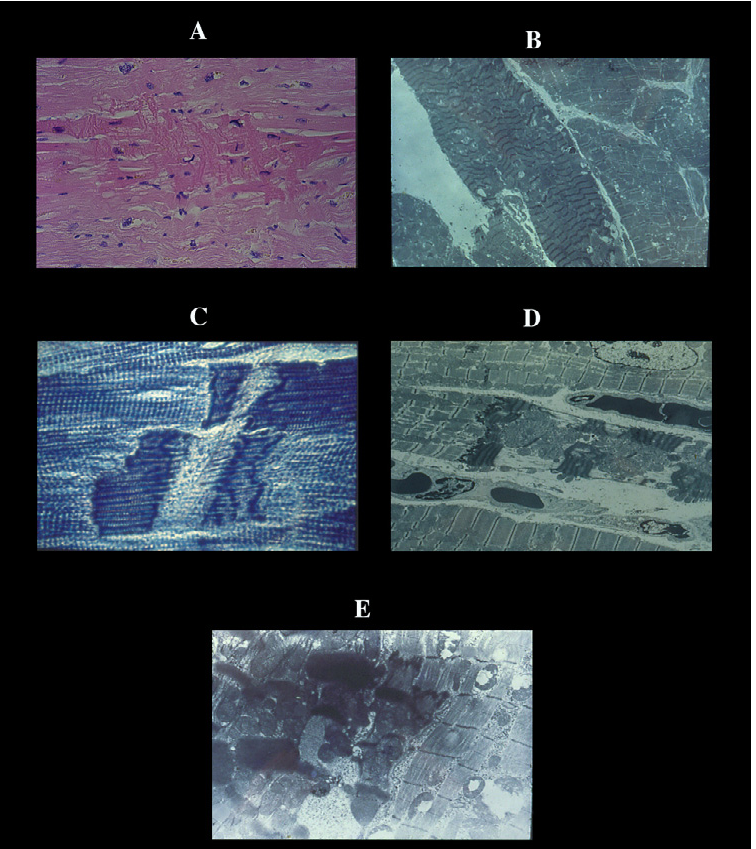
Coagulative myocytolysis or contraction band necrosis or catecholamine necrosis (CN). Pancellular lesion involving the whole myocardial cell. A) histological view of a CN focus. B) ultrastructural hypercontraction with extremely short sarcomeres and highly thickened Z lines and focal myofibrillar rhexis. C) rupture of a hypercontracted myocell. EM view of pathological bands (D) formed by segments of hypercontracted and coagulated sarcomeres (intravenous infusion of catecholamines in dogs).

**Figure 3:**
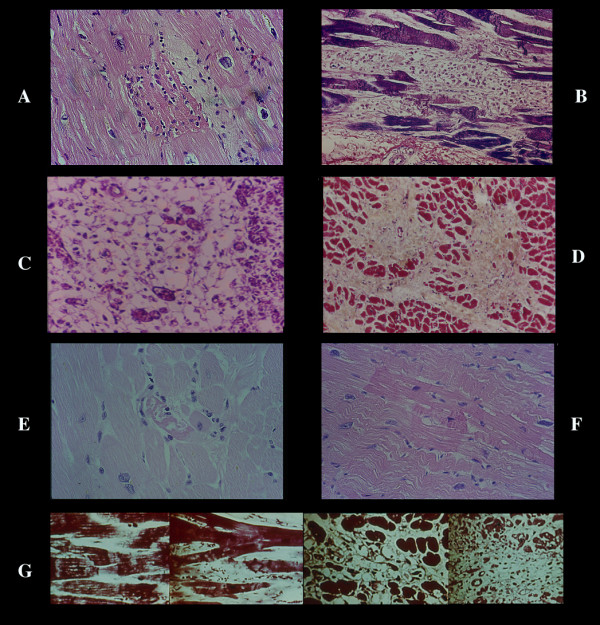
Repair process of CN. A) early monocytes infiltration which later, becomes extensive especially in large necrotic foci (B). It can be misinterpreted as lymphocytic myocarditis. This macrophagic reaction results in empty sarcolemmal tubes with numerous macrophages often loaded with lipofuscin and normal intramural vessels (C). The end result is a focal or plurifocal or confluent fibrosis (D). Microfocal fibrosis as result of necrosis of few myocells (E) can be confused with proliferation of collagen matrix. F) wavyness of normal myofiber around hypercontracted elements. G) all stages of CN in human pheochromocytoma.

The term "contraction bands" needs a more precise definition. Apart from the changeable "physiological bands" in relation to the normal contraction cycle and beside catecholamine necrosis, other "pathological bands" must be considered:

a. *Paradiscal bands*, part of catecholamine necrosis as a unique band of 10–15 hypercontracted sarcomeres adjacent to an intercalated disc, in an otherwise normal cell. This band does not show any rhexis, macrophagic reaction or other changes and may involve two adjacent myocytes from both sides of the same disc and may appear as a clear or dark band (Fig. 4). Already visible after 5 minutes from catecholamine infusion, the paradiscal bands correspond to the "zonal lesion" described in experimental hemorrhagic shock and prevented by betablocker. It is unclear if this change is a reversible one since in our experimental and human material a reaction of repair process was never seen. The clear band could represent a rebuilding of few damaged sarcomeres in a normally working myocyte.

**Figure 4:**
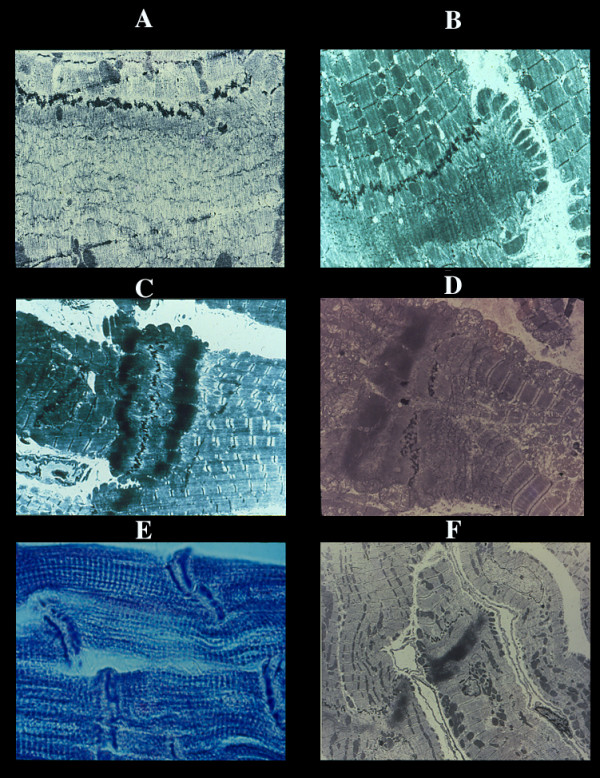
CN. Paradiscal lesion. Always associated with the pancellular lesion, is already visible in experimental intravenous infusion of catecholamines within 5 minutes (pancellular within 10 minutes). It is formed by a unique band of hypercontraction involving 10–15 sarcomeres adjacent to an intercalated disc. The major part of the myocell is normal and this lesion shows ultrastructurally (A) a clear aspect without rhexis, thin myofibrils and Z lines (rebuilding of normal sarcomeres?) or as a band with different grade of density (B-D), often involving two myocells (C). The dense band can be see histologically (E). An hypercontracted "center" (F) induces wavyness of normal adjacent myocells seen by EM.

b. *Cutting edge lesion *i.e. a 0.5 millimeter layer of hypercontracted sarcomeres along the cut margin of living myocardium (biopsy, surgical sample, heart excised at transplantation); an artefact not to be confused with catecholamine necrosis (Fig. 5A,B)

**Figure 5:**
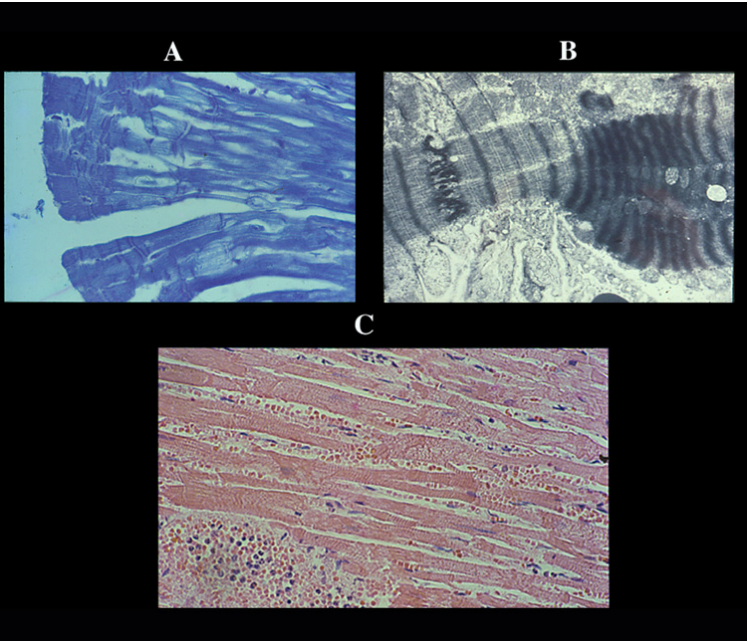
Cutting edge lesion which involves a layer of 0.2–0.5 mm along the cut margin of a living myocardium (biopsies, surgical samples, heart excised at transplantation). A) histological aspect in heart excised at transplantation and B) ultrastructural pattern in dog. C) reflow or reperfusion necrosis characterized by CN plus massive interstitial hemorrhage never seen in other human and experimental conditions.

c. *Reperfusion injury*. From an experimental model of a) temporary coronary occlusion followed by reflow or b) long lasting coronary occlusion, the "wave front phenomenon" has been proposed [6], namely the expansion of a primary infarct established within one hour after occlusion of left circumflex coronary artery and limited to subendocardial layer and posterior papillary muscle [7]. Such an expansion includes the initial infarct with stretched necrotic myocytes, surrounded first by a large zone of "contraction band necrosis" associated with massive hemorrhage and externally by macrophagic reaction and reparative process (Fig. 5C).

This model has been erroneously considered to mimic human infarct. In 200 fatal, acute infarct cases, without any attempt of revascularization, resuscitation and fibronolytic therapy, the ischemic/reperfusion changes were never observed and wavefront expansion was due to nonhemorrhagic catecholamine necrosis, always present both in continuity with the central ischemic necrosis and in normal surrounding myocardium as well as in myocardium not related to the occluded artery. By left circumflex permanent occlusion for 10,18,40 and 60 minutes and temporary occlusion far 10 minutes followed by a 5 minute reperfusion in dog, we tested location and extent (number of foci and necrotic myocytes × 100 mm^2^) of catecholamine necrosis. The latter was present with a similar extent in ischemic and non ischemic myocardium being independent from amount of flow calculated by radioactive microspheres. Both myonecrosis and frequently associated ventricular fibrillation were prevented by beta-blocker.

For a better understanding of the meaning of catecholamine necrosis in cardiology, its presence and extent were quantified in different conditions (Table 2). The catecholamine myotoxicity was significantly higher in conditions with an adrenergic overtone than in normal controls dead from accident. In the latters with a short survival some damage likely due to an agonal release of interstitial catecholamines (not seen in instantaneous death) was found.

**Table 2:** Catecholamine myocardial necrosis – Frequency and extent in different conditions

**Source**	**Sudden/unexpected death**			**Brain hemorrhage**		**Transplant heart**	**AIDS**	Congestive heart failure*	**Cocaine**	**Head trauma**		**Electro cution**	**Carbon monoxide intoxication**
	**Coronary**		**Changes**										
**Number cases**	**25**		**34**	**27**		**46**	**38**	**144**	**26**	**45**		**21**	**26**

	**no infarct+**			**survival ≤ 1 day >**						**survival ≤ 1 hour > 1**			
			
	**21**	4*		**14**	**13**					**26**	**19**		

Catecholamine necrosis													
Present	15	4	34	12	12	39	25	126	11	1	8	1	3
Foci	27 ± 10	29 ± 10	3 ± 1	16 ± 5	37 ± 14	36 ± 9	4 ± 2	2 ± 0.3	4 ± 1	0.5	12 ± 6	8	1 ± 0.5
Myocytes	185 ± 48	1717 ± 698	34 ± 16	26 ± 29	108 ± 134	262 ± 47	13 ± 5	11 ± 2	11 ± 4	35	21 ± 12	46	5 ± 2
Cross band	2	1	8	9	4	14	19	65	11	1	8	1	3
Alveolar	11	2	16	3	6	17	6	25	-	-	-	-	-
Healing	2	1	10	-	2	8	-	36	-	-	-	-	-

*Silent infarct in apparently normal subjects. **Hearts excised at transplantation.

Foci and myocells quantified × 100 mm^2 ^(standard error).

The conclusion was that catecholamine necrosis is an important signal of adrenergic stress [3,4] particularly in in sudden coronary death, (too often interpreted as synonymous of infarct), in which the unique acute lesion found was catecholamine necrosis in about 80% of cases while in 20% a "silent" infarct associated with catecholamine necrosis was detected. These figures are in agreement with clinical studies in resuscitated people [3].

**3. **The third damage consists in a disappearance of myofibrils with increasing myocardial cell vacuolization, edema and small mitochondria without any reaction (macrophages, inflammatory elements). This change (*colliquative myocytolysis*) was seen in about 40% of acute infarct cases, around vessels and in subendocardium in myocardial layers preserved by ischemic necrosis (Fig. 6). Its maximal frequency and extent was in congestive heart failure independently from the underlying disease (Tab. 3). This damage indicates failure of the myocardium, when other rare causes of vacuolization are excluded.

**Figure 6:**
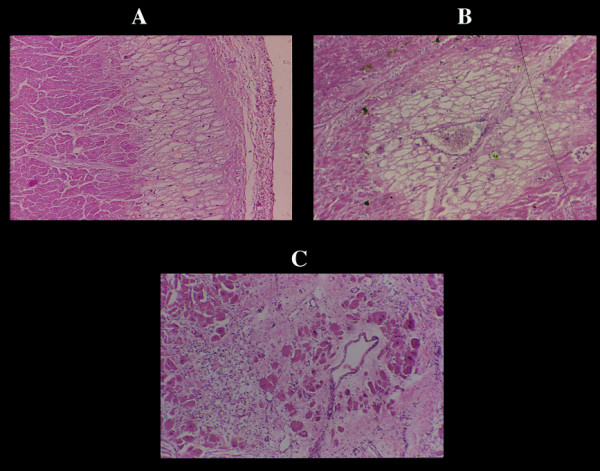
Colliquative myocytolysis associated with acute myocardial infarct. The lesion is confined in layers of the subendocardial myocardium (A) or around functioning vessels (B). These layers are preserved by the infarct necrosis as shown (C) in a perivascular myocardial layer around a vessel in an old infarct without congestive heart failure.

**Table 3:** Frequency and grade of colliquative myocytolysis in different conditions

**Source**	**Sudden/Unexpected death**		**Transplanted**	Congestive heart failure*			**Brain**	**AIDS**	**Cocaine**	**Carbon**	**Head**	**Electrocution**
	**Coronary**	**Chagas**	**hearts**	**CHD**	**DCM**	**VPT**	**hemorrhage**			**monoxide**	**trauma**	
**Cases**	**25**	**34**	**46**	**63**	**63**	**18**	**27**	**38**	**26**	**26**	**45**	**21**

**Colliquative myocytolysis grade**												
0	19	28	33	1	3	1	26	33	26	26	45	21
1	6	6	11	14	28	2	-	3	-	-	-	-
2	-	-	1	39	28	14	1	2	-	-	-	-
3	-	-	1	9	4	1	-	-	-	-	-	-

Grading: 0 = absent, 1 few or smalls group of "empty" myocytes, 2 less and 3 more the 50% of involved myocytes in the inner half of cardiac wall

As a matter of fact, the recognition of different forms of "functional" myonecrosis, which diverge totally in term of structural pathology and molecular/ion biology, denies the assemblage of acute coronary syndromes as a unique etiopathogenetic entity; and helps in interpreting the evolutive phase of each one syndrome as sequence of events and their own causes and mechanisms. For example, a recent consensus [8] included all types of necrosis (coagulation necrosis, contraction band necrosis, apoptosis) measleading our understanding on what a myocardial infarct is

## Myocardial Disarray

**Figure 7:**
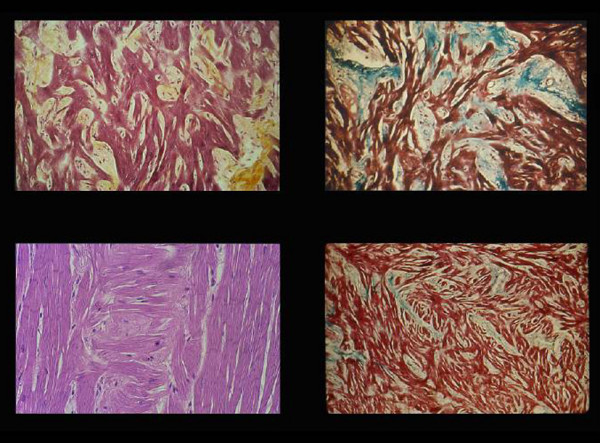
Myocardial disarray. Different aspects (A-D) with increased interstitial fibrosis.

In discussing the myocardial cause of cardiac arrest, myocardial disarray is another pattern to be considered. It consists of a structural disorganization of the myocardium in which myocytes, instead of their usual parallel arrangement for a correct cardiac pump function, assume a star-like disposition with elements oriented obliquely or perpendicular to each other and joined by short, generally hypertrophic myobridges with interconnecting myofibrils and increased interstitial fibrosis (Fig. 7). This architectonic disorder without evidence of myocellular primary damage is visible in some specific zones of normal hearts at the site of directional change (apex, interventricular septum) of myocardial bundles suggesting "junctional nodes" to help contraction. Furthermore myocardial disarray has been observed around scars, in congenital malformed hearts, lentiginosis, Friedreich's ataxia, Turner's syndrome, hyperthyroidism and overall in hypertrophic cardiomyopathy. Its correlation with the adrenergic system has been suggested by human and experimental data. We studied frequency, extent of myocardial disarray in zones normally uninvolved, in conditions with and without adrenergic hypertone (Table 4). A significant increase in frequency and extent of myocardial disarray was documented in "adrenergic overtone" conditions and it correlated with frequency and extent of catecholamine necrosis. An interesting observation was the absence of myocardial disarray in transplanted hearts of patients dead in the first week after surgery in contrast to its presence in longer survivors [9]. The conclusion was that myocardial disarray, more frequent than originally supposed, may be linked with adrenergic stress and should be diagnosed in time due to its asynergic and arrhythmogenic effect leading to ventricular fibrillation.

**Table 4:** Frequency and number of sites with myocardial disarray in different conditions

**Source**	**Sudden/unexpected death**		**Brain hemorrhage**	**Transplanted hearts**				**Congestive heart failure**	**AIDS**	**Cocaine**	**Carbon monoxide**	**Electtrocution**	**Head trauma**
	**Coronary**	**Chagas**		**<7**	**7–30**	**31–365**	**>365**						
**Cases**	**25**	**34**	**27**	**9**	**10**	**13**	**14**	**144**	**38**	**26**	**26**	**21**	**45**

**Disarray sites**													
0	13	25	12	9	4	6	6	124	34	22	26	21	45
1	2	4	-	-	1	-	2	10	-	4	-	-	-
2	-	4	4	-	1	-	-	5	-	-	-	-	-
3	2	-	1	-	-	1	1	2	2	-	-	-	-
4	1	1	3	-	3	-	1	-	1	-	-	-	-
5	2	-	1	-	-	3	-	3	-	-	-	-	-
6	-	-	1	-	-	-	-	-	-	-	-	-	-
7	2	-	-	-	-	2	1	-	-	-	-	-	-
8	3	-	5	-	-	1	3	-	-	-	-	-	-

Sites = 8 myocardial samplings from anterior, lateral posterior left and right ventricles and anterior and posterior interventricular septum. Samples were taken from zones which normally do not have focal disarray.

## Myocardial Asynergy – Cardiac Arrest

Asynergy or dissinergy means a permanent or temporary, global or zonal contractile dysfunction. It may happen in any condition (coronary heart disease, cardiomyopathies, myocarditis, congenital malformation, etc) with the impression, that, no matter what the underlying disease is, asynergy is linked mainly with the morphofunctional damages previously described. Accordingly, the two apparently opposite patterns of non-functioning but viable myocardium secondary one to chronic ischemia (*hibernating myocardium *which return to function following revascularization) and the other to reperfusion (*stunned myocardium *able to refunction after hours, day or weeks) could be explained as a reversible form of relaxed or contracted phase; an assumption derived by experimental permanent coronary occlusion with flaccid myocyte paralysis and catecholamine venous infusion with hypercontraction. Any clinico-pathological correlation is irrealistic since reversibility means no damage of structures which return to function; their temporary blockage is likely at a molecular/ionic level difficult to see histologically and detectable only by immuno-histochemical or more sophisticated techniques at one condition: to sample the dysfunctioning myocardium (serial sections) and discriminate unrelated terminal changes.

In the previous review on coronary collaterals we questioned the existence of chronic ischemia and in the present one we question the existence of reperfusion necrosis in human pathology; suggesting possible alternative etiopathogenetic mechanisms, in which the autonomic nervous system may play an essential role. Agreement exists that in coronary heart disease (CHD), the starting point is zonal hypokinesis (denervation?). May the latter aggravate (akinesis-paradoxical bulging by increased intraventricular pressure) with consequent block by compression of vessels within the non-functioning myocardium, ending in infarct necrosis? Increased contractility by nervous reflexes of surrounding, normal myocardium to compensate the loss of contractility of infarcted myocardium, may result in catecholamine necrosis and ventricular fibrillation (cardiac arrest). In sudden coronary death catecholamine necrosis seems the trigger of ventricular fibrillation. However, in pheochromocytoma in man and in experimental infusion of catecholamines with widespread myocardial lesions ventricular fibrillation does not occur. Only when injected in one coronary artery (unpublished data), noradrenaline produces its typical myocardial necrosis and ventricular fibrillation. The question, therefore, is whether medial neuritis (i.e. lympho-plasmacellular inflammation involving nerves of the tunica in media in CHD) may be the trigger of local noradrenaline release. Similarly in sudden non coronary death in cases with myocarditis associated with catecholamine necrosis (as, for instance, in silent Chagas disease) we should investigate if myocarditis involves intramyocardial innervation. Other possibilities exist in relation to toxic substances or a direct brain/heart relationship with a release in excess of noradrenaline within the myocardium. On the other site, colliquative myocytolysis, not seen in sudden cardiac death, may indicate an acute or subacute or chronic congestive heart failure following an acute infarct or any other cardiac disease.

## Target of Ultrasound Diagnosis: Present and Future

Information on composition and structure of myocardial tissue could be of major importance to better characterize the onset and progression of several myocardial diseases in both clinical and research setting. The use of ultrasounds techniques for this purpose is not new, since first applications date back to 40 years ago [10]. Its theoretical background is represented by the fact that ultrasound interacts differently with abnormal as compared to normal myocardium. However, an ideal technique is still far from ready for clinical use. Different methods, using both qualitative and quantitative approaches have been suggested during the last decades.

### - Qualitative methods

The direct identification of specific abnormalities by the visual inspection of both M-mode and B-mode echocardiograms is the simplest technique used to study the characteristics of myocardial tissue. An increased intensity of the echocardiographic signal has been reported some weeks following anteroseptal myocardial infarction [10]. The same authors were also able to demonstrate a strong correlation between intensity of the signal and presence of scar tissue on surgical or post-mortem evaluation [11,12]. Color encoded digital processing of images proved to further improve the dynamic range of echocardiographic information [13]. In addition, the simple combination of increased acoustic reflectance and reduced end-diastolic thickness has been shown to represent a simple and reliable predictor of the scarred, asynergic myocardial segments which do not improve in function after revascularization [14]. In particular, it has very recently been confirmed that a diastolic wall thickness of ≤ 0.6 cm on baseline echocardiography can exclude the presence of significant viability with a negative predictive accuracy similar to that of dobutamine stress echocardiography [15].

### - Quantitative methods

The biological basis of quantitative methods that have been introduced for the ultrasound tissue characterization is represented by the possibility that individual structural components of the myocardium can influence its acoustic properties in different physiologic and pathologic conditions [16]. These methods are essentially represented by radiofrequency (integrated backscatter) [17,18] and echocardiographic gray level (videodensitometry) analysis [19,20]. In particular, integrated backscatter analyzes the unprocessed radiofrequency signal returning from the myocardium, whilst videodensitometry bases on the conversion of analogic conventional ultrasonic images into a digitized form which allowing quantitative analysis of the ultrasonic myocardial texture. Both techniques [21,22] have been used in experimental or stress-induced myocardial ischemia to detect changes in the ultrasound property of the myocardium. In the setting of acute myocardial infarction, a dramatic reduction of the cyclic variation of integrated backscatter has been demonstrated in the infarct area [23]. Moreover, it was found that myocardial infarcts show an increase in integrated backscatter values and a loss of the cardiac cycle dependent variation in backscatter [24]. This characteristics may be of help in differentiating them from viable tissue [25] that shows preserved cyclic variation of the backscatter signal despite the reduction in wall motion [26]. Differentiation of viable from nonviable tissue has been recently attempted using wavelet transform analysis [27,28], a technique based on breaking up a signal into shifted (translation) and scaled (stretching or compressing) version of a mother wavelet signal [29], to calculate texture energy.

Despite promising preliminary remarks, the pathophysiological background as well as the effective clinical value of ultrasound tissue characterization remain to be defined. In particular, it is expected that more standardized approaches, that will be available in the very near future from digitized technologies, can be of help in allowing comparison of the results from different laboratories.

### - Tissue Doppler Imaging

During a prolonged coronary artery occlusion, myocardial necrosis progresses from endocardium toward epicardium as a wave-front phenomenon [30]. Anatomic-pathological studies revealed the great heterogeneity of the reperfused myocardium that contains a variable amount of necrosis surrounded by a viable but transiently stunned epicardium [31]. This structural and functional heterogeneity complicates the interpretation of wall motion abnormalities by conventional echocardiography. Tissue Doppler imaging is a relatively recent ultrasound technique enabling quantification of intramural myocardial velocities by detection of consecutive phase shifts of the ultrasound signal reflected from the contracting myocardium [32]. Main interest of the technique for the myocardial tissue characterization is associated with its ability to differentiate transmural from nontransmural myocardial infarction and thus to assess myocardial viability [33].

Further improvements in both qualitative and quantitative imaging techniques are expected in the near future. This may provide a powerful tool to make information on biochemical composition and physiological state of the myocardial tissue easily available in clinical practice.

## Competing interests

The author(s) declare that they have no competing interests.

## Authors' contributions

Prof. Giorgio Baroldi contributed to the conception and organization of this review and to the final comments.

Dr. Riccardo Bigi and Dr. Lauro Cortigiani summarized the use of ultrasound techniques in atherosclerotic plaque imaging
